# Peak modulation in multicavity-coupled graphene-based waveguide system

**DOI:** 10.1186/s11671-016-1791-0

**Published:** 2017-01-05

**Authors:** Jicheng Wang, Xiaosai Wang, Hongyan Shao, Zheng-Da Hu, Gaige Zheng, Feng Zhang

**Affiliations:** 1 School of Science, Jiangsu Provincial Research Center of Light Industrial Optoelectronic Engineering and Technology, Jiangnan University, Wuxi, 214122 China; 2 Key Laboratory of Semiconductor Materials Science, Institute of Semiconductors, Chinese Academy of Sciences, 912, Beijing, 100083 China; 3 School of Physics and Optoelectronic Engineering, Nanjing University of Information Science & Technology, Nanjing, 210044 China

**Keywords:** Plasmonically induced transparency, Graphene-based waveguide, Multiple peak modulation

## Abstract

Plasmonically induced transparency (PIT) in a multicavity-coupled graphene-based waveguide system is investigated theoretically and numerically. By using the finite element method (FEM), the multiple mode effect can be achieved, and blue shift is exhibited by tunable altering the chemical potential of the monolayer graphene. We find that the increasing number of the graphene rectangle cavity (GRC) achieves the multiple PIT peaks. In addition, we find that the PIT peaks reduce to just one when the distance between the third cavity and the second one is 100 nm. Easily to be experimentally fabricated, this graphene-based waveguide system has many potential applications for the advancement of 3D ultra-compact, high-performance, and dynamical modulation plasmonic devices.

## Background

Electromagnetically induced transparency (EIT) refers to a quantum destructive interference phenomenon between two excitation pathways with a sharp transparency window [1–3]. Based on the EIT effect, many potential applications in quantum information, ultrafast optical switching, nonlinear optics, slow light effect have been proposed [4–6]. However, the practical applications of EIT effect were restricted because of the difficulty with demanding experimental conditions [7]. With theoretical analysis and experimental operation, EIT-like response occurred in the near-field coupling and the phase coupling, was observed to address the problem [8]. Especially, as a novel phenomenon analogous to EIT, plasmonically induced transmission (PIT) [9–15] based on surface plasmon polaritons (SPPs) has been researched in highly integrated metal-based nanoscale plasmonic devices due to its unique properties for allowing the propagation of light to overcome the diffraction limit and manipulating light at subwavelength scales [16–19]. Nevertheless, these metallic plasmonic structures are mainly applied and achieved in the visible regions. The abilities to dynamically control the PIT response remain the significant challenges.

Graphene, a single-layer carbon atom arranged in a honeycomb lattice, has been vigorously investigated as a promising platform for plasmonic result from its remarkable characteristics, such as strong mode confinement, low loss, and active tunability [20]. In particular, by applying extra gate voltage, magnetic fields, and chemical doping, the surface conductivity of graphene can be changed, which gives an increasing development on tunable plasmonic devices [21–24]. Considering the unique properties, graphene can also support propagation of SPPs, which are polariton modes of photon and electron density waves along a conductor and dielectric interface. Graphene surface plasmon polaritons (GSPPs) can not only be excited for a wide range of wavelengths from near-infrared to terahertz (THz) region [25], but also display lower propagation loss due to the high carrier mobility of graphene at room temperature [26]. Moreover, it is easier to control the propagation of GSPPs by altering the chemical potential of graphene without re-fabricating the structure. The highly tunable plasmonic graphene nanoresonators at mid-infrared have been observed by Brar et al. using infrared microscopy [27]. A two coplanar graphene strips coupling system supported on substrates [28], and a graphene-based resonator-coupled waveguide system [29], the tunable multiple layer graphene-based absorber [30, 31] have been researched. However, the modulation of PIT peaks with multi-cavity-coupled graphene sheet system has not been discussed.

In this paper, we investigate a novel 3D graphene-based plasmonic waveguide system. This system is composed of one guided graphene waveguide (GGW) coupled to one to three graphene rectangle cavity (GRC). The multimode PIT phenomenon has been observed in our proposed system. Two transmission dips exist with one GRC while two PIT peaks appear in the dip-position of the transmission spectra with two GRCs. The increasing number of the GRC placed above the GGW introduces the multi-PIT peaks. In addition, the multimode PIT effects can be dynamically controlled by varying the chemical potential and the coupling distance. Moreover, the modulation of PIT peaks with three cavities is investigated in detail. For the advancement in 3D ultra-compact high-performance PIT devices, our work may have potential applications in the PIT-peaks modulation, slow light and optical switching.

## Methods

**Fig. 1:**
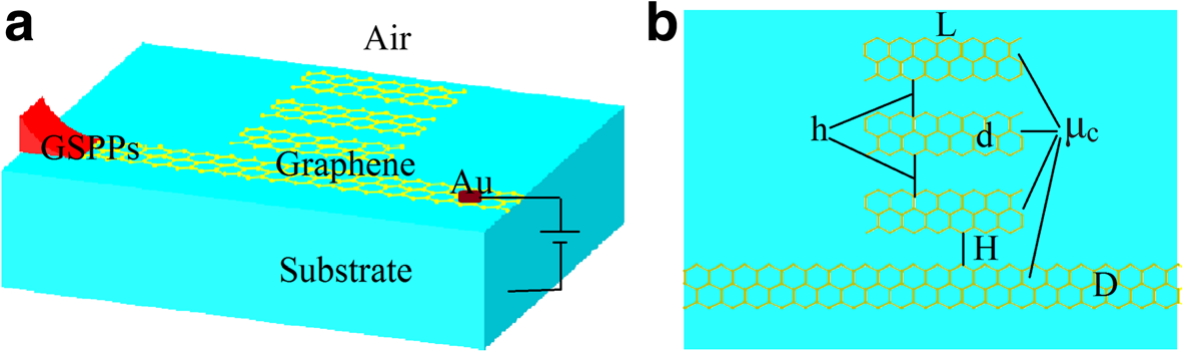
**a** Schematic illustration of the three cavities coupled graphene waveguide system. **b** The planform of the monolayer graphene with the chemical potential *μ*

A schematic of the structure of the designed tunable peak modulation devices based on three GRCs waveguide system is shown in Fig. 1. The incident wave propagates along the graphene waveguide to the right port while some parts of the GSPPs couple to the graphene cavities. The width and length of the three same cavities are *d* and *L*, respectively. The width of the waveguide is *D*. Here, *D* and *d* are fixed to be 10 nm, *L* is 100 nm. In addition, *H* and *h* represent the waveguide-cavity coupling distance and the cavity-cavity coupling distance, respectively. Chemical potential *μ*_*c*_ is applied to the graphene waveguide and cavities. We carry out the three-dimensional numerical simulations by using the FEM [32]. The Kubo formula has governed the surface conductivity of graphene *σ*_*g*_ including the intraband and interband transition contributions [20]:

1 $$ {\sigma}_{\mathsf{intra}}=\frac{i{e}^2{\mu}_c}{\pi {\hslash}^2\left(\omega +i{\tau}^{-1}\right)}, $$

2 $$ {\sigma}_{\mathsf{inter}}=\frac{i{e}^2}{4\pi \hslash } \ln \left[\frac{2{\mu}_c-\left(\omega +i{\tau}^{-1}\right)\hslash }{2{\mu}_c+\left(\omega +i{\tau}^{-1}\right)\hslash}\right]+\frac{i{e}^2{k}_BT}{\pi {\hslash}^2\left(\omega +i{\tau}^{-1}\right)} \ln \left[ \exp \left(-\frac{\mu_c}{k_BT}\right)+1\right]. $$

It depends on chemical potential *μ*_*c*_, photon frequency *ω*, momentum relaxation time *τ*, and temperature *T*. Here, *e* represents the electron charge. In our numerical simulations, the incident wave is at mid-infrared where the influence of interband transition in graphene can be negligible. In this case, the optical conductivity can be simply given by:

3 $$ {\sigma}_g\left(\omega \right)=\frac{i{e}^2{\mu}_c/\pi {\hslash}^2}{\omega +i{\tau}^{-1}}. $$

The following equation shows the equivalent permittivity of graphene [33]:

4 $$ {\varepsilon}_g=1+\frac{i{\sigma}_g{\eta}_0}{k_0\varDelta }, $$

where the wave vector in vacuum *k*_0_ = 2*π/λ* and the intrinsic impedance of air *η*_0_ ≈ 377 Ω. The monolayer graphene support TM-polarized SPPs is only in consideration for our analysis. The dispersion relation of this TM GSPP surface wave follows the equation:

5 $$ {\beta}_{\mathrm{GSPP}}={k}_0\sqrt{1-{\left(\frac{2}{\eta_0{\sigma}_g}\right)}^2}, $$

where *β*_GSPP_ is the propagation constant of GSPPs. Defined as *n*_eff_ = *β*_GSPP_ */k*_0_, the effective index of GSPPs features the capacity for confine SPPs on graphene. Obviously, from the equations above, the Re*(n*_eff_ *)* increases for a fixed wavelength as the chemical potential *μ*_*c*_ decreases, which intends the GSPPs are better confined at lower chemical potential. Importantly, with a slight change in chemical potential, the Re(*n*_eff_) varies greatly, which leads to the design of dynamically tunable peak modulation devices. The transmission of GSSPs with one graphene cavity satisfies the resonant condition:

6 $$ 2\mathrm{R}\mathrm{e}\left({\beta}_{\mathrm{GSPP}}\right)L+2{\varphi}_1=2m\pi . $$

Here, *φ*_1_ is the additional phase [34]. *m* standing for the order of resonant mode (RM), is an integer. *L* is length of the cavities. Moreover, it should be noted that we assume the substrate to be air in our 3D simulations for simplification. The reason is given in the following part.

## Results and Discussion

**Fig. 2:**
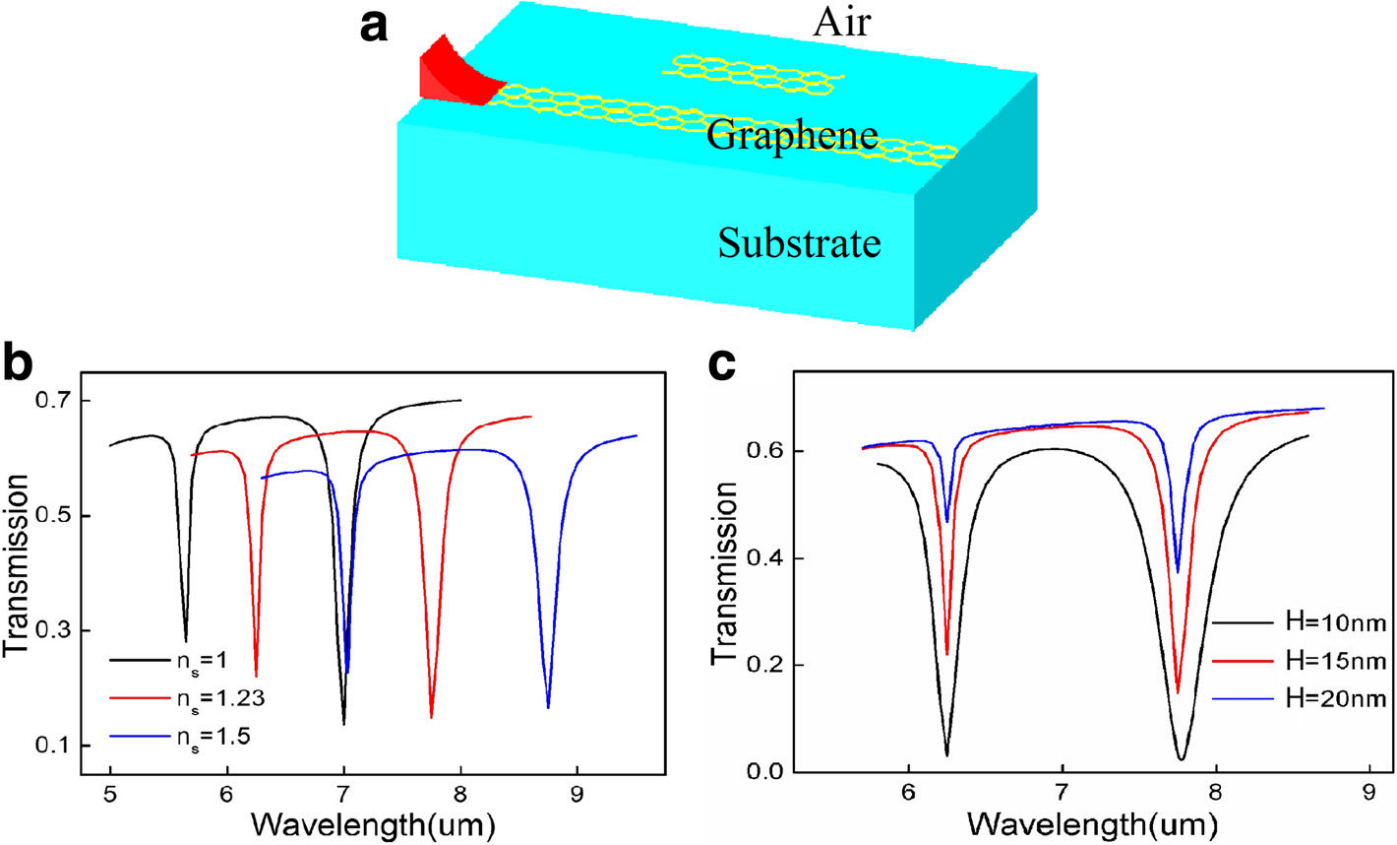
**a** Schematic illustration of one cavity coupled graphene waveguide system. **b** The transmission spectra with different substrates. **c** The transmission spectra with different waveguide-cavity coupling distances

As shown in Fig. 1, GSPPs can be coupled to the GRCs when the incident wave propagates along the GGW. In order to fully understand the properties of the proposed waveguide system, we investigate the transmission characteristics of the GGW with one graphene cavity as shown in Fig. 2. Figure 2a shows the schematic with the same geometrical parameters as Fig. 1a. For future experimental design purpose, we also give the transmission spectra with different substrates, in which *n*_*s*_ = 1 and *n*_*s*_ = 1.23 represent the substrate to be air and SiO_2_, respectively. From Fig. 2b, we can see that the different substrates (such as silica, silicon carbide) only lead to a read shift in the transmission spectra. The transmission with different coupling distances *H* is shown in Fig. 2c. As the distance increases, the waveguide-cavity coupling gets weaken meanwhile more energy transport to the right port and thus the value of the dips becomes larger.

**Fig. 3:**
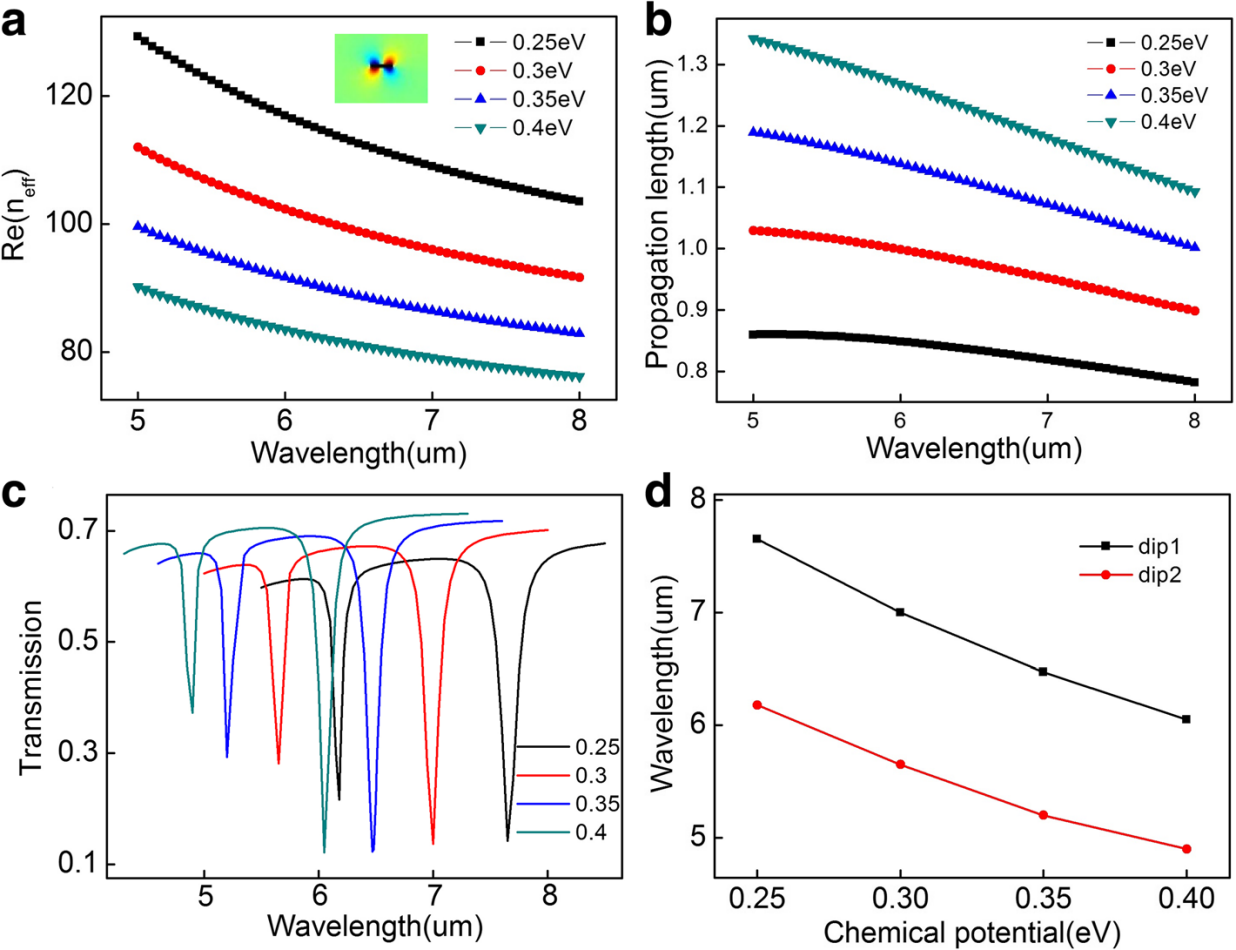
**a** The effective refractive index and **b** the propagation length of the waveguide mode as a function of the incident wavelength with different chemical potential, **c** the transmission spectra of the system under different chemical potential, **d** the transmission dips as a function of chemical potential

The effective refractive index of the waveguide mode in relation to the incident wavelength is depicted in Fig. 3a. The insert figure gives the sectional view of the mode distribution propagating along the waveguide. It is apparent that the value of decreases as the chemical potential increasing, which agrees well with theoretical equations mentioned above. Figure 3b indicates the propagation length increases with the chemical potential increases. In addition, as the incident wavelength increase, the propagation length has a decrease. The dependence of the transmission spectra on the chemical potential is shown in Fig. 3c. Moreover, a blue shift of the transmission dip1 (*λ*_1_ = 7 μm at *μ*_*c*_ = 0.3 eV) and dip2 (*λ*_2_ = 5.65 μm at *μ*_*c*_ = 0.3 eV) is observed. Subsequently, Fig. 3d provides the two-dips-position as a function of chemical potential which emerge a good linear relationship.

**Fig. 4:**
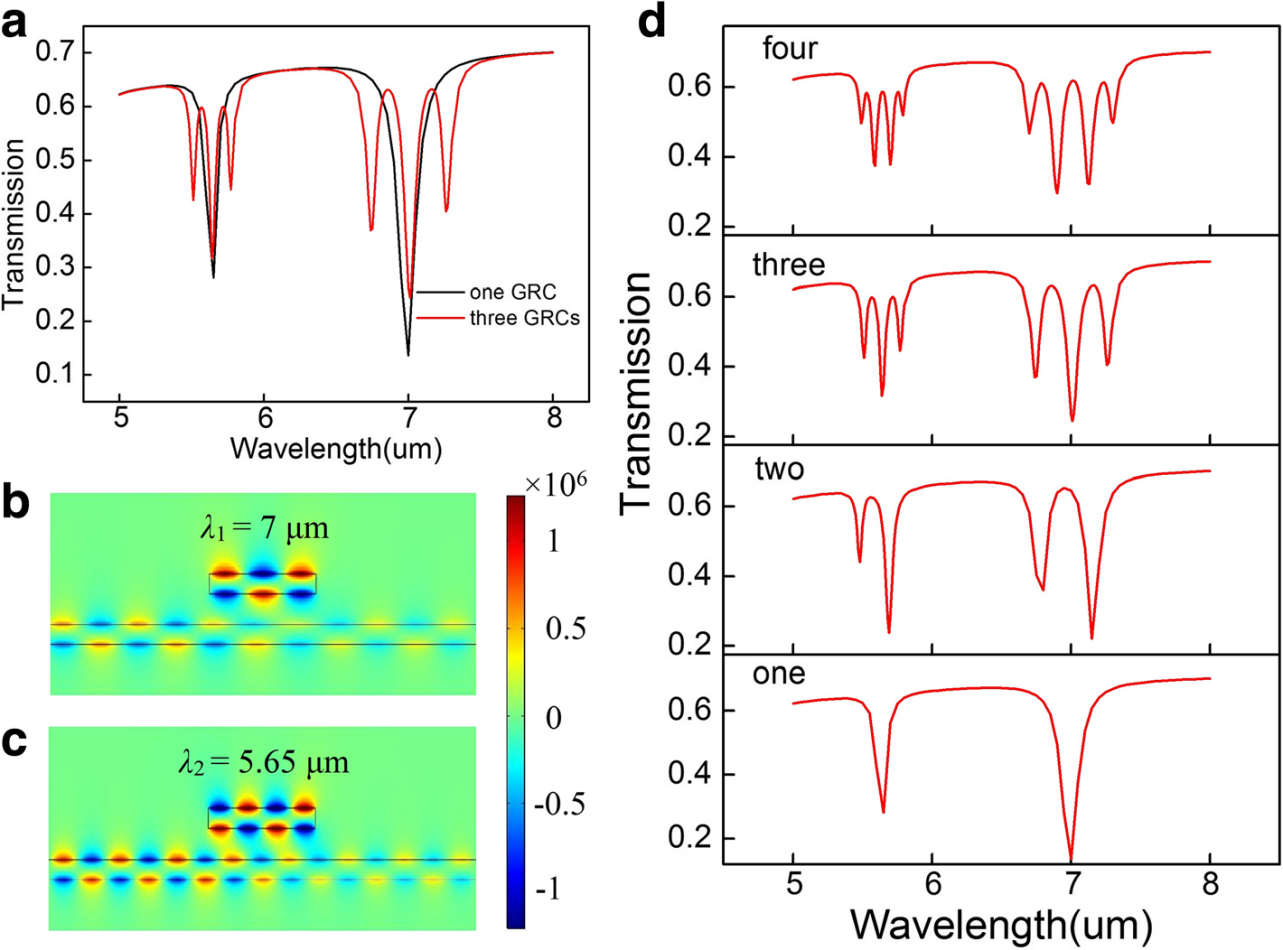
**a** The transmission spectra with one GRC (*black line*) and three GRCs (*red line*), **b** and **c** the counter profiles of the magnetic field distributions |*H*_*z*_| at transmission dips *λ*_1_ = 7 μm and *λ*_2_ = 5.65 μm, **d** the transmission spectra with one to four GRCs at chemical potential *μ*_*c*_ = 0.3 eV

Based on the transmission characteristics of the graphene waveguide with one cavity, we investigate the properties of the system with multi-cavities. The black line in Fig. 4a presents two obvious transmission dips at the incident wavelengths *λ*_1_ = 7 μm and *λ*_2_ = 5.65 μm can be generated in the transmission spectra in the condition that the one GRC is in resonance with the waveguide. There are, respectively, two obvious transmission PIT peaks at the two resonant wavelengths due to the near-field coupling between the three cavities, and the waveguide as depicted in Fig. 4a with red line. The counter profiles of the magnetic field distributions |*H*_z_| with one cavity in the two resonant wavelengths are shown in Fig. 4b and c. At *λ*_1_ = 7 μm, the third order RM exists in the GRC, while *λ*_2_ = 5.65 μm satisfies the fourth order resonant condition. At the same time, most parts of the GSPPs cannot confine to the GRC and thus propagate to the right port of the GGW. The PIT effect can be shown by the transmission spectrum with multi-cavities under the chemical potential *μ*_*c*_ = 0.3 eV in Fig. 4d. The increasing number of the GRCs gives rise to the PIT peaks. When only one rectangle cavity is placed above the waveguide, it behaves as a Fabry-Perot (FP) resonator due to the near-field coupling. Adding another cavity to the system, the PIT window appears which is attributed to the additional cavity-cavity coupling. The PIT peaks are located at *λ*_1_ = 6.95 μm and *λ*_2_ = 5.6 μm, respectively. It indicates that two GRCs generate one PIT peak. Subsequently, we investigate the transmission properties of the waveguide system with three and even four graphene cavities. As shown in Fig. 4d, three cavities lead to two peaks and four cavities correspond to three PIT peaks. Therefore, the number of the PIT peaks is always one less than the cavity numbers as we expect. We can also see that the value of the PIT peaks is almost the same. This is because the incident wavelengths do not satisfy the resonant condition so the GSPPs propagate along the waveguide without coupling losses. What is more, the maximum value of the transmission is less than 1 because of the scattering losses and the absorption of the materials.

**Fig. 5:**
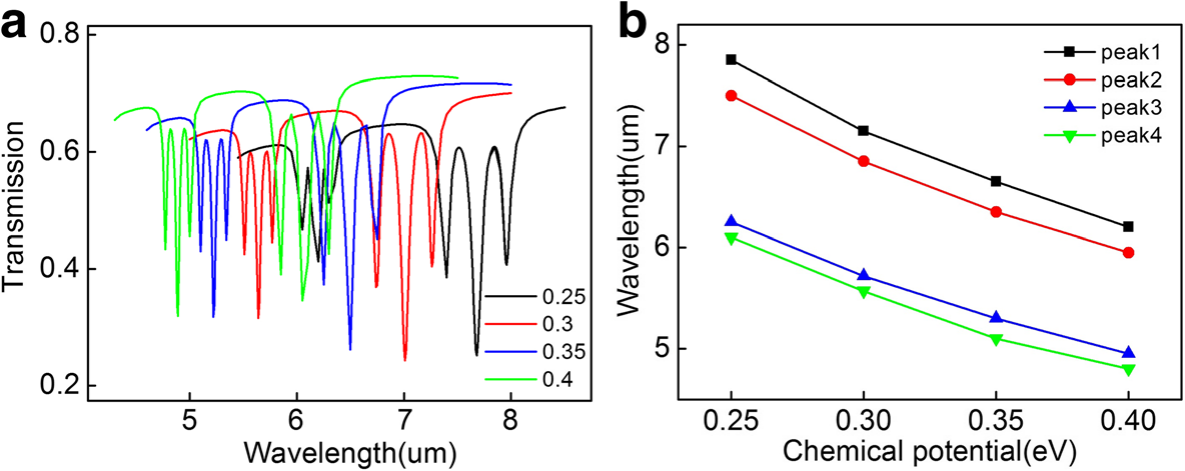
**a** The transmission spectra with different chemical potential. **b** Peak wavelengths as a function of chemical potential

As the surface conductivity of graphene depends on the chemical potential can determine the propagation of the GSPPs, we can achieve the dynamic tunability of the PIT peak by changing the chemical potential of cavities and waveguide. The transmission spectrum with different chemical potential is shown in Fig. 5a. It is obvious that with the increase of the chemical potential, the PIT peaks happen to a blue shift which can be explained by the resonant condition between the graphene cavities. The resonant wavelength satisfies *λ* ∝ (2*π* ^2^ *ℏcD*/*α*_0_ *μ*_*c*_)^1/2^ ∝ (1/*μ*_*c*_)^1/2^ [35, 36]. As a result, a blue shift is obtained in the transmission peaks with chemical potential increasing. The four PIT peaks in one transmission spectrum are named as peak1 ~ 4 from right to left in turn. A good linear relationship between peak wavelength and chemical potential is exhibited in Fig. 5b.

**Fig. 6:**
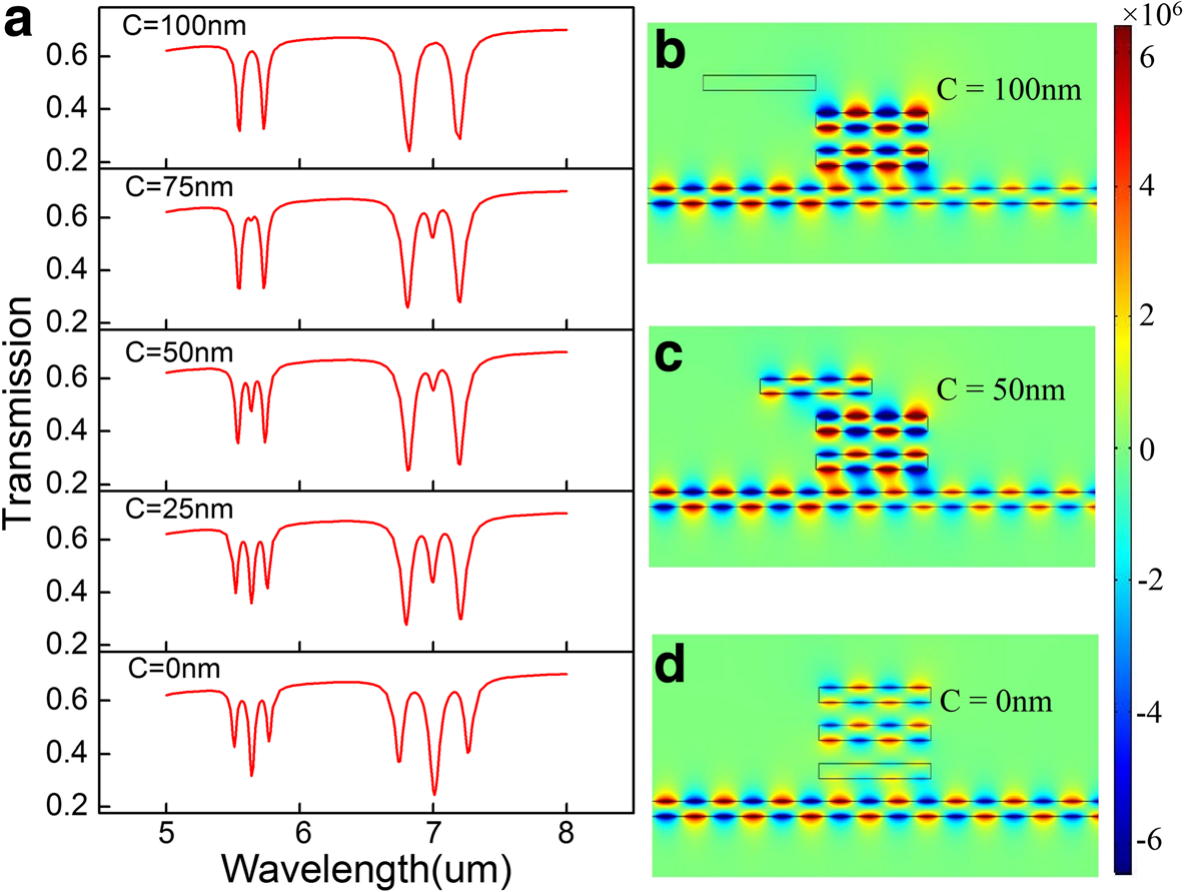
**a** The transmission spectra with different coupling distance *C*, **b**–**d** the counter profiles of the magnetic field distributions |*H*_*z*_| at peak *4* with *C* = 0, 50, 100 nm, respectively

To further explore the influence of the rectangle cavities to the PIT peaks, we change the position of the third cavity with other parameters remain the same. Here, *C* is used to describe the distance between the center of the second cavity and the center of the third one. It is apparent that the cavity coupling between the second and the third cavity is gradually weakened as *C* increases. Therefore, the number of PIT peaks in the two PIT windows decrease to one at *C* = 100 nm as depicted in Fig. 6a. In order to illuminate the resonant characteristics of the PIT effect, we give the magnetic field distributions in *z* direction with different coupling distances *C* in Fig. 6b–d at incident wavelength *λ* = 5.55 μm. As shown in Fig. 6d, there is no cavity coupling between the second and the third cavity resulting from the distance *C* is too far to confine. Thus, the peak number reduces to 1 in good consistent with the transmission spectrum seen in Fig. 6a.

## Conclusion

In conclusion, our work particularly has focused on the peak modulation of the multicavity-coupled graphene-based waveguide system by using FEM. One graphene cavity leads to a transmission dip while two cavities generate a PIT window in the transmission spectra. The number of the PIT peaks is always one less than the number of the cavities. Moreover, not only a blue shift can be achieved in transmission spectra with the increasing of chemical potential, but also a good linear relationship between peak wavelength and chemical potential is obtained. The increasing coupling distance *C* can lead to the subdued cavity-cavity resonance and eventually achieve the peak modulation. We believe the proposed structures, easily to be operated, may promise the applications for tunable multiple mode filters and slow light devices.

## Abbreviations

EIT: Electromagnetically induced transparency
FEM: Finite element method
GGW: Guided graphene waveguide
GRC: Graphene rectangle cavity
GSPPs: Graphene surface plasmon polaritons
PIT: Plasmonically induced transparency
THz: Terahertz
TM: Transverse magnetic
